# The impact of immigration on microbial community composition in full-scale anaerobic digesters

**DOI:** 10.1038/s41598-017-09303-0

**Published:** 2017-08-24

**Authors:** Rasmus H. Kirkegaard, Simon J. McIlroy, Jannie M. Kristensen, Marta Nierychlo, Søren M. Karst, Morten S. Dueholm, Mads Albertsen, Per H. Nielsen

**Affiliations:** Centre for Microbial Communities, Department of Chemistry and Bioscience, Aalborg University, Fredrik Bajers Vej 7H, DK-9220 Aalborg, Denmark

## Abstract

Anaerobic digestion is widely applied to treat organic waste at wastewater treatment plants. Characterisation of the underlying microbiology represents a source of information to develop strategies for improved operation. Hence, we investigated microbial communities of thirty-two full-scale anaerobic digesters over a six-year period using 16S rRNA gene amplicon sequencing. Sampling of the sludge fed into these systems revealed that several of the most abundant populations were likely inactive and immigrating with the influent. This observation indicates that a failure to consider immigration will interfere with correlation analysis and give an inaccurate picture of the growing microbial community. Furthermore, several abundant OTUs could not be classified to genus level with commonly applied taxonomies, making inference of their function unreliable and comparison to other studies problematic. As such, the existing MiDAS taxonomy was updated to include these abundant phylotypes. The communities of individual digesters surveyed were remarkably similar – with only 300 OTUs representing 80% of the total reads across all plants, and 15% of these identified as non-growing and possibly inactive immigrating microbes. By identifying abundant and growing taxa in anaerobic digestion, this study paves the way for targeted characterisation of the process-important organisms towards an in-depth understanding of the microbiology.

## Introduction

Biogas production from the anaerobic digestion of organic waste is increasingly being implemented as an alternative renewable energy source. This change is driven by the need for clean energy as well as improved economy of wastewater treatment plants by making them into net energy producers^1^. Methane gas production from organics is mediated by the tightly coupled synergistic activities of complex microbial communities and is essentially covered by four sequential stages: hydrolysis, fermentation, acetogenesis and methanogenesis. The anaerobic digestion process is generally robust, but occasionally reactors experience operational problems such as foaming events and periods of low efficiency or failure^2–4^. A better understanding of the underlying microbiology will facilitate optimisation of the biological processes, and consequently, the microbiology has been widely studied using various approaches with both lab-scale and full-scale systems^5–7^.

Understanding the ecology of anaerobic digesters, and how it relates to system function, first requires the identification of the active and abundant microorganisms and subsequent linkage of their identity to their functional roles^8^. Several 16S rRNA gene amplicon based studies have shown that there appears to be a set of abundant microorganisms, common to similarly operated anaerobic digesters, that are stably present over time^6, 7, 9, 10^. This is also known for other biological processes, such as wastewater treatment plants^11^ and the human digestive system^12^. Furthermore, other studies have revealed process temperature, substrate composition, and ammonia concentrations as important factors in the shaping of the microbial community composition. However, in anaerobic digesters a large part of the observable microbial community might originate from dead or inactive cells arriving with the influent biomass from which DNA persists. Hence, the observed microbial community dynamics will not truly reflect the changes in process performance or stability. This can lead to spurious correlations and false conclusions^11^. In an attempt to mitigate the problem of DNA from inactive cells influencing microbial analysis, molecular techniques have been developed to remove or bind the extracellular DNA prior to cell lysis^13, 14^. However, the complex matrix of anaerobic digester sludge samples will likely lead to problems with unwanted chemical reactions and limited penetration of the light used in the process^14^. Hence, an alternative approach is to monitor the microbial composition in the influent to identify organisms whose abundance is likely maintained by immigration^11, 15–17^.

Associating phylogeny with function is essential for understanding the ecology of these systems. However, a substantial proportion (67%^9^ to 73%^7^) of sequences obtained in previous 16S rRNA gene amplicon surveys of anaerobic digesters were not classified to the genus level with the commonly applied taxonomies: such as SILVA, RDP and Greengenes^18–20^. Furthermore, biases associated with DNA extraction, primer coverage and differences in the taxonomy applied for classification^21, 22^, greatly hampers cross-study comparisons. Hence, only by using well-defined standard methods and the same curated database for taxonomic classification across the field, it is possible to make meaningful cross-study comparisons and robust biological conclusions^23^. Standardisation has been established for activated sludge from wastewater treatment plants with the MiDAS protocols^21^ and the curated MiDAS taxonomy^22^, but is currently lacking for anaerobic digestion.

Another approach to study community composition in anaerobic digesters is metagenomic sequencing^24–28^. However, this approach is currently hampered by the limited number of genomes in the reference databases, which results in a poor classification of reads and contigs^27^, and there is thus a pressing need for populating the genome databases with the relevant genomes before such an approach is truly meaningful^28, 29^.

The aim of this study was to identify the abundant and growing organisms in full-scale anaerobic digester systems, fed waste activated sludge, using 16S rRNA gene amplicon sequencing. The survey included 32 Danish full-scale reactors located at 20 wastewater treatment plants over a six-year period (>300 samples), including both mesophilic and thermophilic reactors, and represents the most comprehensive study of full-scale systems to date. Comparison of read abundances in the digester sludge, and the corresponding influent primary and surplus sludge, was used to identify immigrating populations and to provide an assessment of the growing populations in the anaerobic digesters. Furthermore, having identified the abundant populations present in the anaerobic digesters, we have performed a manual curation of the SILVA taxonomy for the most abundant operational taxonomic units (OTUs), many of which were poorly classified with existing databases. By providing genus level classifications for all abundant taxa, researchers in the field will be able to link the identity with the accumulated knowledge regarding their population dynamics and ecophysiology. The comprehensive list of the microorganisms enriched in anaerobic digesters will also form the foundation for prioritising the effort in getting high quality genomes from key members of the microbial communities from metagenomic binning.

## Results

### Characteristic of the sampled anaerobic digesters

More than 300 samples were collected from 32 full-scale digesters at 20 wastewater treatment plants in Denmark over a period of 6 years (2011–2016). The sampled reactors represent mesophilic (~37 °C) and thermophilic (~55 °C) processes running mainly on primary sludge and surplus activated sludge (approx. 50:50% in relative amount of organic matter). The reactors have reported ammonium levels in the range of 500–3000 mg/L, acetate concentrations of 0.5–20 mmol/L, alkalinity levels of 0.01–0.5 mmol/L, pH of 7.1–8.5, reactor volumes of 1300–6000 m^3^ and sludge retention times of 10–55 days. The plants in Fredericia and Næstved have mesophilic reactors with a thermal hydrolysis pre-treatment (THP), the type of pre-treatment in both cases are CambiTHP^TM^ installations.

### Community structure: Archaea

**Figure 1:**
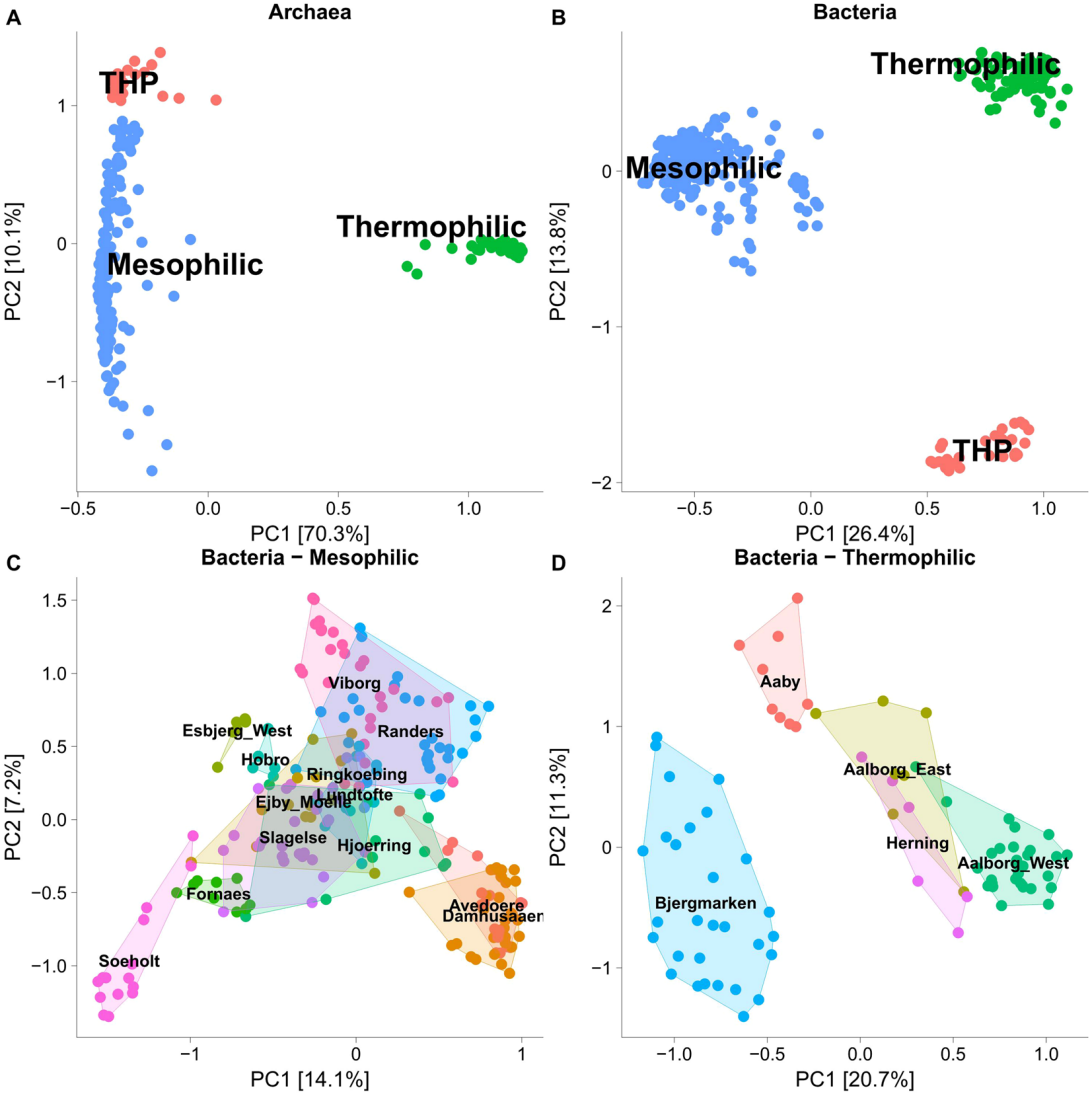
Principal component analysis of the microbial communities in ADs, highlighting samples by process type information ( mesophilic,  thermophilic,  mesophilic with thermal hydrolysis pretreatment (THP)). (**A**) the separation of archaeal communities coloured by process type, (**B**) the separation of bacterial communities coloured by process type, (**C**) The bacterial communities of mesophilic plants coloured and labelled by plant location, (**D**) The bacterial communities of thermophilic plants coloured and labelled by plant location.

**Figure 2:**
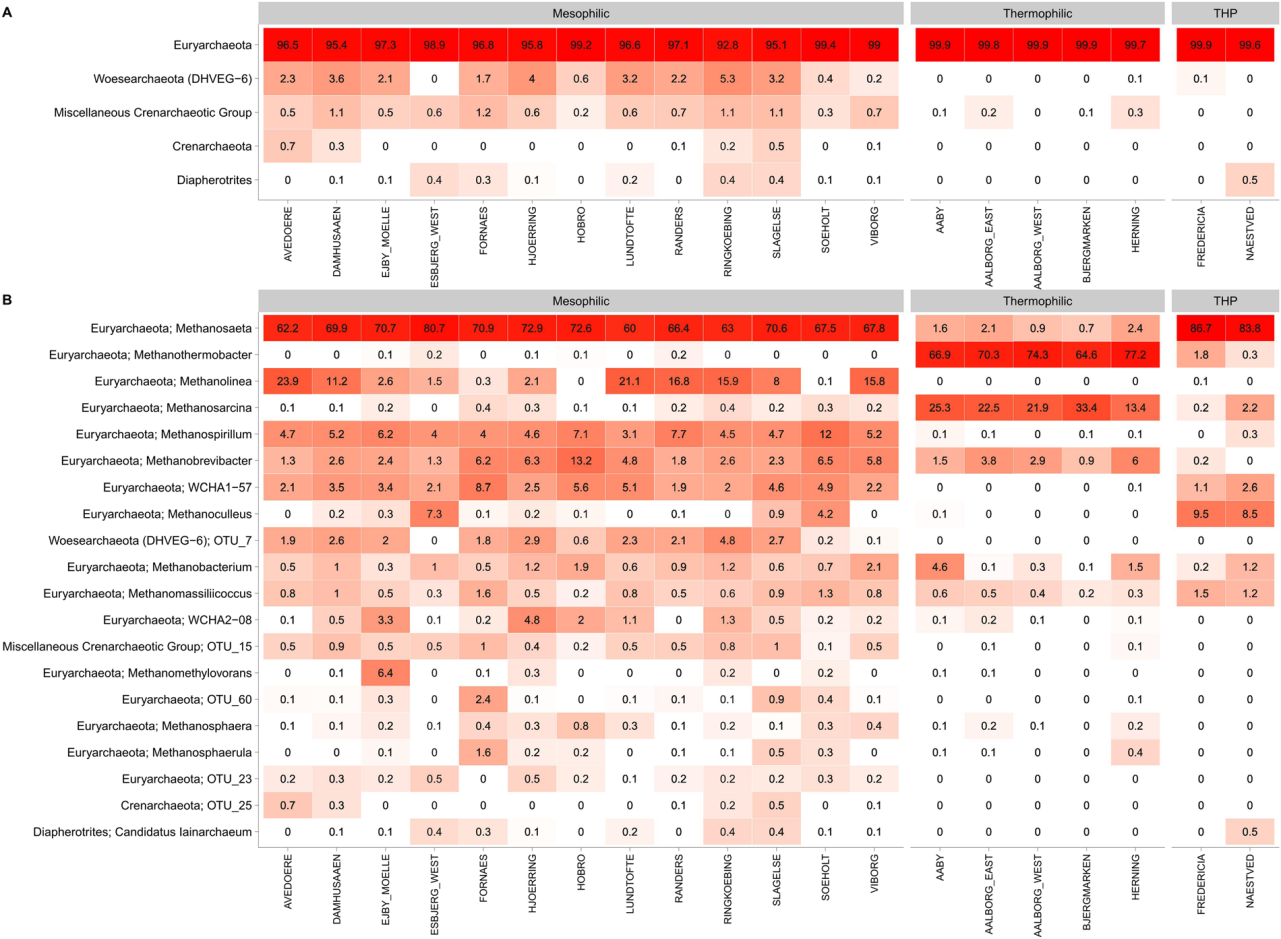
(**A**) Heatmap of the 5 most abundant archaeal phyla. (**B**) Heatmap of the 20 most abundant archaeal genera in the anaerobic digesters. When no genus level classification is available the OTU number is given. The phylum level classification is shown for all genera. Data based on 32 AD reactors (1–4 per plant) analysed 2–23 times. The mean read abundance is shown for each plant. The taxa are sorted by mean read abundance across the plants at the respective phylogenetic level (phylum, genus).

The archaea were targeted with archaea-specific primers amplifying the V3-5 regions of the 16 S rRNA gene. The resulting quality filtered sequencing data were subsampled to 10 000 reads per sample, giving more than 3 million reads in total. There were 169 OTUs (97% sequence identity), spanning 8 phyla, which constituted at least 0.1% in a single sample. Principal component analysis revealed that the thermophilic and mesophilic reactors formed very distinct archaeal communities (Fig. 1A). Euryarchaeota was by far the most dominant archaeal phylum making up 93–100% of the archaeal reads in each sample (Fig. 2A). The acetoclastic methanogenic genus *Methanosaeta* dominated the sequencing libraries of the mesophilic reactors (60–80% of the reads), followed by a variety of hydrogenoclastic methanogenic genera such as *Methanolinea*, *Methanospirillum*, *Methanobrevibacter* as well as the WCHA1-57, which was recently renamed *Candidatus* Methanofastidiosa^30^ (Fig. 2B). The mesophilic reactors with thermal hydrolysis pre-treatment were also dominated by *Methanosaeta* (83–87%). The underlying OTUs for the most abundant genera were the same for the different plants (Fig. S1). For the genus *Methanosaeta*, there was one dominant OTU (25–33% relative read abundance) and six additional OTUs in read abundances of each 2–15% in all mesophilic reactors, including those with THP, indicating a substantial diversity within the genus.

The thermophilic reactors were dominated by the hydrogenoclastic methanogenic genus *Methanothermobacter* (64–77% of the reads), followed by the more versatile *Methanosarcina* (13–33% of the reads). The latter is known to perform both acetoclastic and hydrogenoclastic methanogenesis. *Methanobrevibacter* was the third most common methanogen and along with *Methanosaeta*, the only abundant archaeon shared with the mesophilic reactors. However, it was not found in mesophilic reactors with thermal hydrolysis pre-treatment. The underlying OTUs for the two abundant genera were the same for the different plants (Fig. S1). For *Methanothermobacter*, there was one dominant OTU (37–48% relative read abundance) and two less abundant OTUs (6–20%). For *Methanosarcina*, there was one dominant OTU (10–25%) and one less abundant OTU (3–6%). The archaeal community of the thermophilic samples clearly had a lower diversity than the mesophilic samples **(**Fig. 2B & See diversity metrics in Fig. S2).

### Community structure: Bacteria

**Figure 3:**
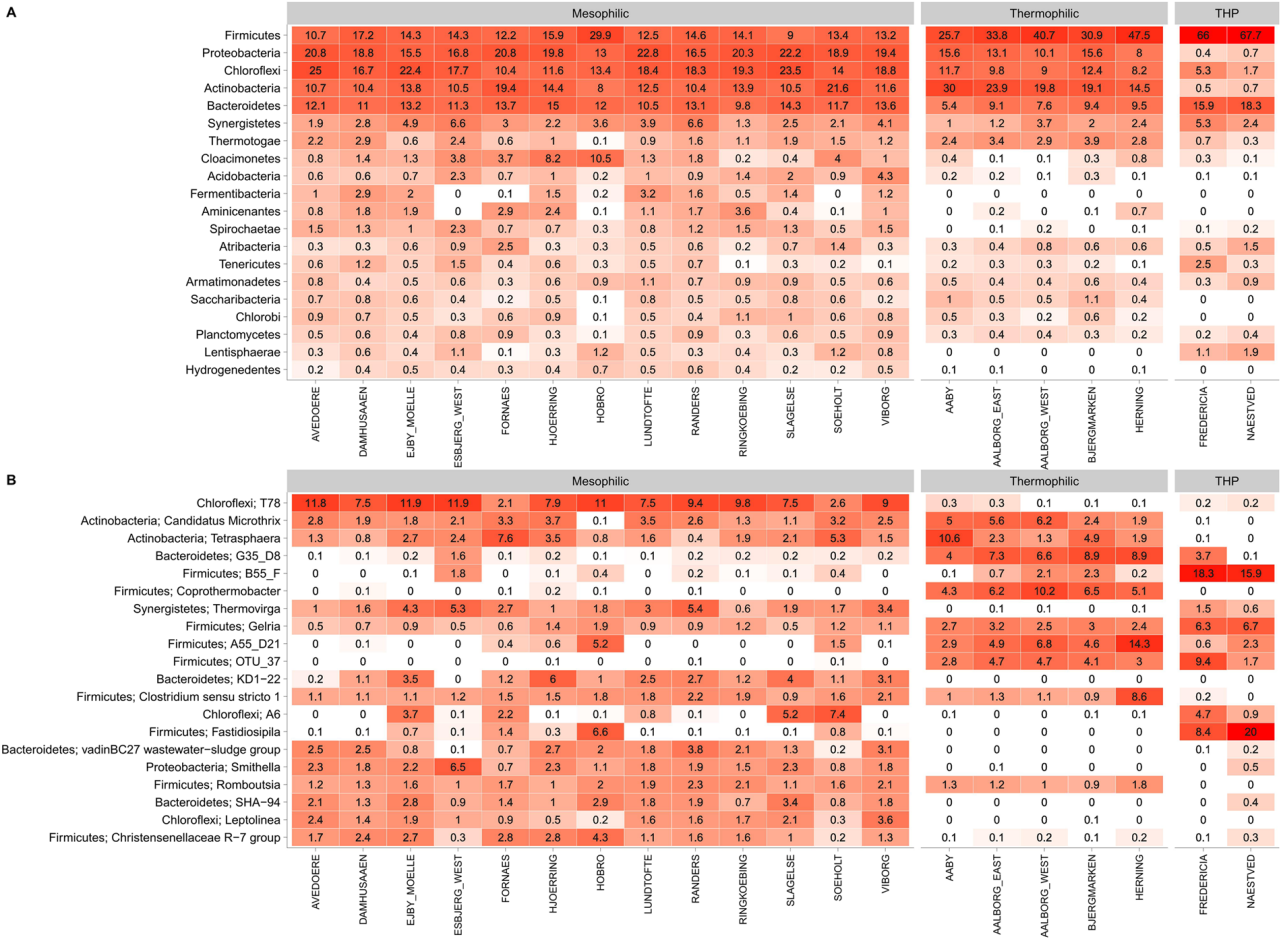
(**A**) Heatmap of the 20 most abundant bacterial phyla. (**B**) Heatmap of the 20 most abundant bacterial genera in the anaerobic digesters. When no genus level classification is available the OTU number is given. The phylum level classification is shown for all genera. Data based on 32 AD reactors (1–4 per plant) analysed 3–30 times. The mean read abundance is shown for each plant. The taxa are sorted by mean read abundance across the plants at the respective phylogenetic level (phylum, genus).

The bacteria were targeted with bacteria-specific primers amplifying the V1-3 regions of the 16 S rRNA gene. The resulting quality filtered sequencing data were subsampled to 10 000 reads per sample giving more than 3 million reads in total. The resulting 5614 OTUs, each making up at least 0.1% of the reads in at least one sample, covered 46 phyla. Principal component analysis revealed that the thermophilic and mesophilic reactors formed very distinct bacterial communities with a separate cluster for reactors with thermal hydrolysis pre-treatment (Fig. 1B). Principal component analysis of the samples within the mesophilic and thermophilic clusters **(**Fig. 1C,D) shows that the overall structure of the microbial communities overlap between some plants during the period. The dominant phyla were Firmicutes, Proteobacteria, Actinobacteria, Bacteriodetes and Chloroflexi (Fig. 3A). Along with the more “well-known” phyla, a few candidate phyla, such as Fermentibacteria (Hyd-24–12), Aminicenantes (OP8), and Atribacteria (OP9), were also observed. Most mesophilic reactors were dominated by the MiDAS genus T78 belonging to Chloroflexi, followed by the genera *Tetrasphaera* and *Candidatus* Microthrix (Fig. 3B). The thermophilic reactors also had a high read abundance of *Tetrasphaera* and *Ca*. Microthrix. However, the mesophilic reactors with thermal hydrolysis pre-treatment did not have a notable read abundance of either of these two genera despite them being present in the surplus sludge (Fig. 3B). This suggests that these genera do not grow in mesophilic digesters, but are coming in with the feed. Supporting this idea is that the underlying OTUs for the most abundant genera were the same for the different plants (Fig. S3). The dominant OTUs in the digesters were generally shared among the plants with similar operation (Fig. S4) and as few as 300 OTUs account for 80% of the reads, which is a metric sometimes defined as the “abundant core” (Fig. S5)^11^.

### Community composition of primary and surplus sludge

The feed for all digesters, except Fredericia, was a mixture of primary sludge settled from influent wastewater and surplus sludge harvested from the activated sludge plant, approximately in an organic mass ratio of 50:50. Fredericia had only surplus sludge. The bacterial community composition was analysed in 121 samples of primary sludge from 14 WWTPs and 137 activated sludge samples from all 24 WWTPs. The overall community structure showed clear clustering of the different sample types, separating primary sludge, surplus sludge, mesophilic, thermophilic and THP reactors (Fig. S6), indicating noticeably different communities. The microbial communities in the primary sludge were very similar in all samples and the most abundant genera were *Streptococcus, Arcobacter* and *Trichococcus* (Fig. S7). The most abundant genera in the surplus sludge were also very similar in most plants reflecting the presence of abundant core species such as *Tetrasphaera, Ca*. Microthrix, and *Ca*. Amarilinum (Fig. S8).

### Survival of influent bacteria in the digesters

**Figure 4:**
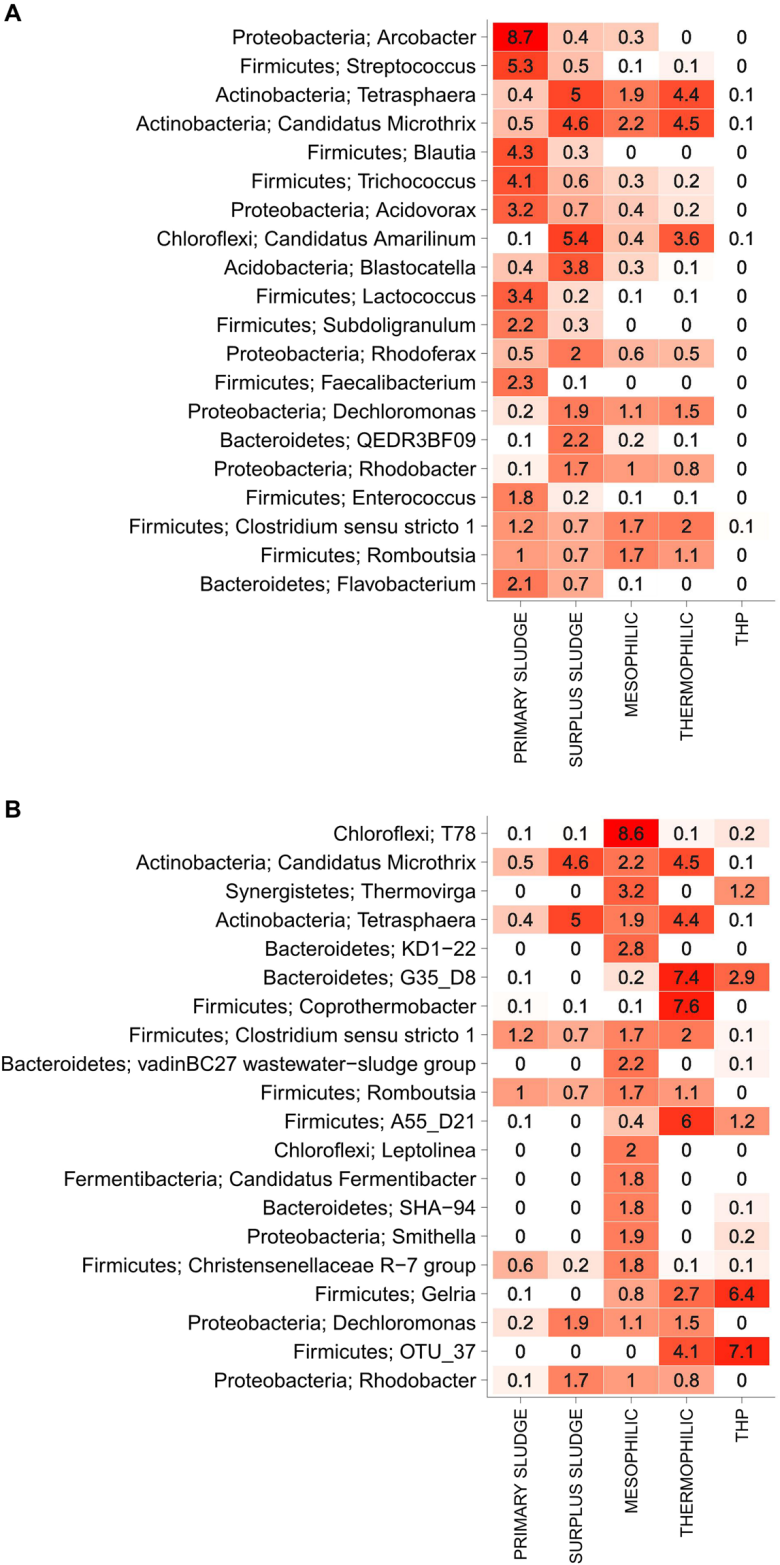
Heatmap of the 20 most abundant bacterial genera (**A**) Taxa sorted by the mean read abundance in the influent (primary and surplus sludge) (**B**) Taxa sorted by the mean read abundance in the anaerobic digesters (mesophilic, thermophilic and THP). The numbers represent mean read abundances for groups with more reactors and more samples (30–279).

**Figure 5:**
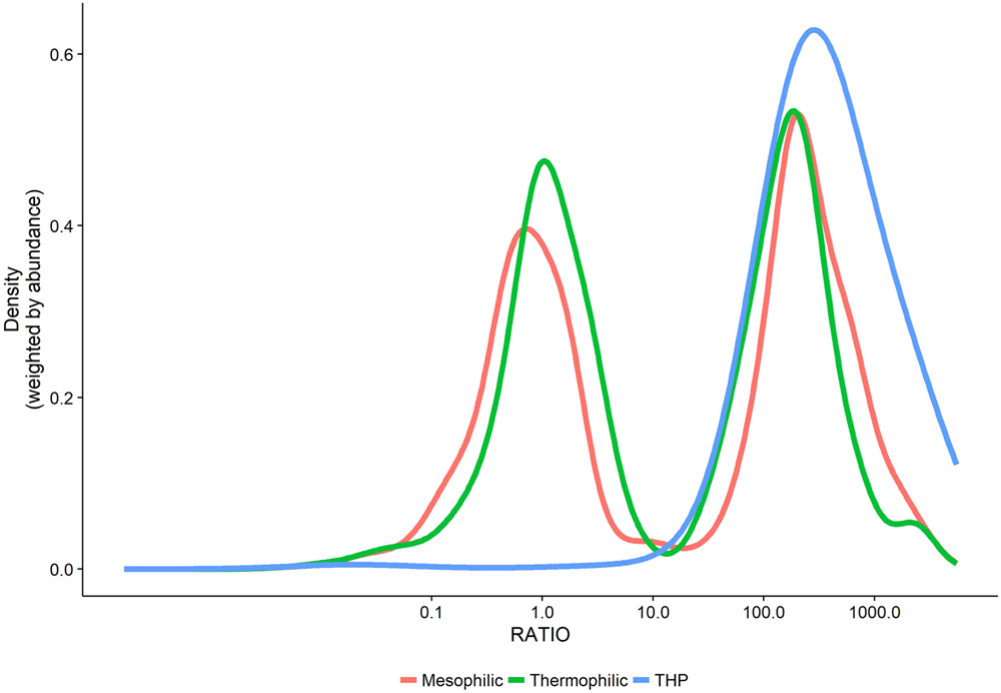
Distribution of ratios between mean OTU read abundance in the anaerobic digesters and the influent streams (primary and surplus sludge) weighted by the read abundance in the anaerobic digesters. Higher ratios mean that the relative read abundance for the OTU is enriched in the digester compared to the primary and surplus sludges, ratios close to 1 or below indicates that the OTU is not enriched in the digester. Mesophilic (), thermophilic (), and mesophilic with thermal hydrolysis pre-treatment ( THP).

Some organisms were present in both of the influent streams and the digesters, whereas others were detected almost exclusively in one of the three sample types (Figs 4 and 5). No overlap was found between the communities in the influent streams and in reactors with THP (Fig. 4). Some organisms, such as *Tetrasphaera*, *Ca*. Microthrix, and *Rhodobacter*, were generally present in both the surplus sludge and the digesters, regardless of process temperature. Other organisms, such as *Arcobacter, Streptococcus*, and *Blautia*, which were the most abundant bacterial genera in the primary sludge, were hardly detected in the digesters.

We tried to assess whether the immigrating organisms tended to die off, survive, or grow in the digesters by calculating the ratios of their mean read abundance in the digester compared to the mean read abundance in the influent streams (Fig. 5, Figs S4, S9 & Table S2). This calculation does not give an exact measure of the growth rate of the individual species as has previously been performed based on detailed mass-balances^11, 17^. However, despite some variability in the sludge retention times and in the fraction of primary and surplus sludge, a clear bimodal distribution of the ratios (Fig. 5) was observed for the mesophilic and thermophilic reactors without the thermal hydrolysis pre-treatment. This indicates that there was a peak for the group of organisms with high ratios that were heavily enriched in the digesters compared to the influent streams, and thus likely growing in the system. The peak with lower ratios include the group of organisms with a read abundance that is unchanged or lower compared to the influent streams and these OTUs are thus likely non-growing or dying off in the digesters. This bimodal behaviour was also seen for the individual plants when samples from influent streams and digesters were analysed (Fig. S9). The bimodal distributions had a split around a ratio of 10 (Fig. 5), indicating that there was a clear difference between organisms growing exclusively in anaerobic digesters and organisms that were dying off or only present because they were fed into the anaerobic digester. The ratio distribution for the THP plants, which were used as a control for reactors without living biomass going into the reactors, had a single peak above 10 supporting that OTUs above this threshold are likely growing in the reactors. Some of the seemingly most abundant organisms in the digesters, such as the genera *Tetrasphaera* and *Ca*. Microthrix, had ratios close to or below one (Table S2), which indicate that they were likely not growing and only present in the digesters by being supplied with the influent streams. However, 203 of the 300 most abundant OTUs had ratios above 10, which indicate that there is a clear shift for microorganisms growing exclusively in the digesters, e.g. the genera *Ca*. Fermentibacter, *Fastidiosipila* and *Coprothermobacter*.

## Discussion

In this study, the microbial communities of 32 full-scale anaerobic digesters at wastewater treatment plants and their influent streams were analysed using 16 S rRNA gene amplicon sequencing to identify the abundant and growing microorganisms of these biotechnologically important systems. Principal component analysis (PCA) revealed that the bacterial communities were distinct for the thermophilic, mesophilic and mesophilic with THP systems (Fig. 1B). The different communities observed in the mesophilic systems, with and without THP, may be partly attributed to the reported higher concentrations of ammonia in the latter (Table S1). These findings are consistent with previous studies which have also shown communities to be clearly influenced by process temperatures and ammonia concentrations^9^. The six-year survey period of the current study indicates that the digester communities at each wastewater treatment plants were relatively stable over time (Fig. S10). Furthermore, more detailed analyses of the taxa revealed that the most abundant organisms were shared between reactors of the same process type (Figs 2 and 3).

Previous studies have also observed abundant organisms that were shared among many anaerobic digester plants of similar operation^6, 7, 9^. This common finding indicates that efforts to characterise the process-important organisms is feasible, with less than 300 OTUs accounting for 80% of the amplicon reads across all plants in the current study. Previous attempts to identify the important genera in anaerobic digesters has been hampered by the lack of taxonomic classification of several abundant organisms with commonly applied taxonomies. As a consequence, previous studies have often focused microbial analysis on OTUs with taxonomic classification to high levels, such as phylum, order or class^6, 7, 9^, where the link between phylogeny and function is more unreliable^31^. In this study, we have sought to address this problem by updating the MiDAS taxonomy to cover abundant genus level taxa in full-scale anaerobic digesters^32^, along with abundant organisms previously identified in activated sludge^22^. Application of the updated taxonomy in this study gave genus level classification for 78% and 97% of all the bacterial and archaeal reads, respectively. Of the bacterial OTUs within top 300 (“abundant core”) the ones with MiDAS specific genus classification accounted for 31% of the bacterial reads.

Importantly, a substantial presence of incoming organisms or their DNA in the community of the assessed digesters was observed in this study (Fig. 5), indicating that some of the seemingly most abundant organisms were related to influent streams rather than growing. To assess the source and activity of abundant organisms, we performed the microbial analysis on the primary and surplus sludge and calculated the ratio of their read abundance in these influent streams and the receiving digesters. The ratios indicate if continuous transfer into the system, and/or active growth, maintains an organism’s read abundance. Fifteen percent of the 300 OTUs, which accounted for 80% of the reads, had ratios of one or below. Four of the 25 most abundant genera (Fig. 3B) had low relative read abundance ratios. These included *Tetrasphaera*, *Ca*. Microthrix, *Clostridium sensu strictu 1* and *Romboutsia*; which are all genera that were also seemingly shared among mesophilic and thermophilic reactors but not present in the reactors with THP. The suggestion that some of these do not belong in anaerobic digesters is also supported by what is known about their metabolism e.g. *Ca*. Microthrix is a known aerobe^33^. A similar approach also identified abundant inactive influent organisms in a single anaerobic digester treating surplus sludge^17^. Inactive organisms identified in the previous study, including *Trichococcus, Rhodobacter* and *Thauera*, were also determined to be non-growing in the current study - having ratios lower than one.

The impact of the influent on the observed community causes a multitude of problems for microbial analysis in digesters as it interferes with attempts to establish relationships between microorganisms and process performance. It is worth noting that, despite likely being non-growing, the persistence of the filamentous members of the genus *Ca*. Microthrix, which is well-known to cause bulking and foaming in activated sludge systems, also has been linked to foaming problems in receiving anaerobic digester systems^34, 35^.

The identification of non-growing populations in anaerobic digesters further shortens the list of microorganisms likely most important to the bulk transformations of these systems. The majority of previously characterised growing genera are known to be fermentative organisms; including *Coprothermobacter* ^36^ and *Anaerobaculum* ^37^ in thermophilic systems, and *Thermovirga* ^38^ *, Leptolinea* ^39^ and *Ca*. Fermentibacter^40^ in mesophilic systems. *Smithella* ^41^ and *Gelria* ^42^ represent known acetogens. In general, apart from the influent organisms, abundant genera were generally not shared between thermophilic and mesophilic systems. The exception within the top 25 is the genus *Gelria* – which was present in both mesophilic and thermophilic reactors with a high read abundance and ratio. The genus was originally isolated from a thermophilic methanogenic enrichment^43^. However, the underlying species-level OTUs differ between the mesophilic and thermophilic reactors, indicating that organisms even within the same genus can occupy distinct niches in these systems (Fig. S11). It is an important observation that for a substantial proportion of the abundant genus-level taxa nothing is known of their potential role in these systems. These include the MiDAS taxa T78, B55_F and G35_D8, within the phyla Chloroflexi, Firmicutes and Bacteroidetes, respectively (Fig. 3B), which are obvious targets for future research into the ecology of these systems. Influent populations of the archaeal domain were not assessed.

The dominant Archaea in the mesophilic reactors running on primary and surplus sludge was *Methanosaeta*, with a range of other hydrogenoclastic organisms such as *Methanolinea*, *Methanospirillum, Methanobrevibacter* as well as *Ca*. Methanofastidiosa (WCHA1-57) at lower read abundances. The uncultured *Ca*. Methanofastidiosa is suggested to be restricted to methylated thiol reduction for methane generation as all known genomes lack genes for acetoclastic and CO_2_-reducing methanogenesis^30^. The dominance of *Methanosaeta* in mesophilic digesters is supported by other studies using amplicon sequencing, qPCR and shotgun sequencing^7, 9, 25^.

*Methanothermobacter* and *Methanosarcina* were the dominant methanogens in the thermophilic systems. The difference between the dominant acetoclastic methanogen could be due to process temperature or shorter residence times as both *Methanosaeta* and *Methanosarcina* cover species able to grow across the entire temperature range of operation^44^. Interestingly, *Methanobrevibacter* was also seemingly abundant in the thermophilic reactors, although it is usually considered mesophilic. However, it was not found in mesophilic reactors with thermal hydrolysis pre-treatment, and *Methanobrevibacter* has previously been found in wastewater treatment processes and isolated from faeces^45–47^ – indicating after all that there may be some influence of immigration on archaeal populations in digesters. In addition to a high read abundance of *Methanosaeta*, the mesophilic reactors with thermal hydrolysis pre-treatment also had a high read abundance of *Methanoculleus*. The methanogen *Methanoculleus* has previously been related to elevated ammonium levels, a relationship that was also supported by the high ammonia levels reported for the THP plants in this study (Table S1)^9, 48, 49^.

In this study, we present a comprehensive list of the growing microorganisms of full-scale anaerobic digesters receiving primary and surplus sludge from wastewater treatment plants (Fig. S4). The relatively low number of genera makes the organisms needed to study feasible, and biological informed decisions less complex and more tractable. Standard application of the curated MiDAS database^32^ for anaerobic digester systems, located at wastewater treatment plants, will form an important foundation for future studies of the ecology of these biotechnologically important systems. However, it is important to keep in mind that the list will likely be missing some of the important players due to PCR biases^50^ and that we need primer-free alternatives to get the entire picture of the microbial diversity in anaerobic digesters^51^.

## Materials and Methods

### Sampling

Biomass samples from digesters were obtained 2–4 times a year in the period 2011–2016 from 37 ADs at 21 Danish WWTPs (Supplementary Table S1). For primary sludge, 121 samples from 14 WWTPs were sampled during 3 months in October-December, 2014. Each sample was based on flow proportional sampling collected through 24 h. For surplus activated sludge, 137 sludge samples were obtained from the aeration tank from 23 WWTPs. All samples were homogenised and stored as 2 mL aliquots at −80 °C for DNA extraction.

### DNA extraction

DNA was extracted from biomass samples using the FastDNA® Spin kit for soil (MP Biomedicals, Santa Ana, CA, USA) following the standard protocol, except for a 4-time increase in the bead beating duration- as recommended by Albertsen *et al*.,(2015)^21^. The biomass input volume was 50 µl for AD sludge and 500 µl for primary sludge and activated sludge. Primary sludge samples were first filtered onto 0.2-µm pore size polycarbonate filters and the DNA extracted from these using the same method described for other samples.

### DNA amplification and sequencing

#### Bacterial PCR

The bacterial primers used were 27 F (AGAGTTTGATCCTGGCTCAG^52^) and 534 R (ATTACCGCGGCTGCTGG^53^), which amplify a DNA fragment of ~500 bp of the 16 S rRNA gene (variable regions 1–3). 25 µL PCR reactions in duplicate were run for each sample using 1X Platinum® High fidelity buffer, 100 µM of each dNTP, 1.5 mM MgSO_4_, 1 U Platinum® Taq DNA Polymerase High Fidelity (Thermo Fisher Scientific, USA), 400 nM of each barcoded V1-V3 primer, and 10 ng template DNA. PCR conditions were 95 °C, for 2 min followed by 30 cycles of {95 °C, for 20 s, 56 °C for 30 s, 72 °C for 60 s} and a final step of elongation at 72 °C for 5 min. PCR products were purified using Agencourt AmpureXP (Beckman Coulter, USA) with a ratio of 0.8 bead solution to PCR solution.

#### Archaeal PCR

The archaeal primers used were 340 F (CCCTAHGGGGYGCASCA^54^) and 915 R (GWGCYCCCCCGYCAATTC^54^), which amplify a DNA fragment of ~ 560 bp of the 16 S rRNA gene (variable regions 3–5). 25 µL PCR reactions in duplicate were run for each sample using 1X Platinum® High fidelity buffer, 100 µM of each dNTP, 1.5 mM MgSO_4_, 1 U Platinum® Taq DNA Polymerase High Fidelity (Thermo Fisher Scientific, USA), 400 nM of each V3-V5 primer mix, and 10 ng template DNA. PCR conditions were 95 °C, for 2 min followed by 35 cycles of {95 °C, for 20 s, 50 °C for 30 s, 72 °C for 60 s} and a final step of elongation at 72 °C for 5 min. PCR products were purified using Agencourt AmpureXP (Beckman Coulter, USA) with a ratio of 0.8 bead solution/PCR solution. Illumina adapters and barcodes were added with a second PCR. 2 µL purified PCR product from above was used as template for a 25 µL PCR reaction containing 1X PCRBIO Reaction buffer, PCRBIO HiFi Polymerase (PCR Biosystems, United Kingdom). PCR conditions were 95 °C, for 2 min, 8 cycles of {95 °C, for 20 s, 55 °C for 30 s, 72 °C for 60 s} and a final step of elongation at 72 °C for 5 min.

#### Sequencing

Bacteria and archaea amplicon libraries were pooled separately in equimolar concentrations and diluted to 4 nM. The amplicon libraries were paired-end sequenced (2 × 300 bp) on the Illumina MiSeq using v3 chemistry (Illumina, USA). 10–20% PhiX control library was added to mitigate low diversity library effects.

### Read processing and classification

The read data were processed separately for the bacterial and archaeal analysis.

### Bacteria

The paired end reads for the bacterial libraries were trimmed using trimmomatic^55^ and then merged using FLASH^56^. Bacterial reads were screened for potential PhiX contamination using USEARCH (v. v7.0.1090)^57^. The reads were clustered at 97% similarity using USEARCH and subsequently classified using the RDP classifier^58^ with the MiDAS database. The most abundant bacterial (top 80) OTUs from the mesophilic and thermophilic digesters were used to guide curation of the Silva database NR99 v. 1.23 taxonomy as described previously^22^. The resulting updated MiDAS taxonomy (v. 2.1), covering the abundant organisms of both anaerobic digesters and activated sludge, was applied for all analyses presented in this study.

### Archaea

The size of the archaeal V3-V5 fragments made it unattainable to merge the reads, so only read 1 files were used for the analysis. The reads were trimmed to a length of 275 bp. Archaeal reads were screened for potential PhiX contamination using USEARCH (v. v7.0.1090)^57^. The reads were clustered at 97% similarity using USEARCH and subsequently classified using the RDP classifier^58^ with the MiDAS database. The most abundant archaeal OTUs (top 40) from the mesophilic and thermophilic digesters were used to guide curation of the Silva database NR99 v. 1.23 taxonomy as described previously^22^. The resulting updated MiDAS taxonomy (v. 2.1), covering the abundant organisms of both anaerobic digesters and activated sludge, was applied for all analyses presented in this study.

### Data visualisation

Further processing of the OTU table was carried out in the R environment (v. 3.3.2)^59^ using the R studio IDE^60^ using the ampvis package (v. 1.27.0^21^) for visualisation. The ampvis package wraps a number of packages including the phyloseq package (v. 1.19.1)^61^, ggplot2 (v. 2.2.1), reshape2 (v. 1.4.2)^62^, dplyr (v. 0.5.0)^63^, vegan (v. 2.4–1)^64^, knitr (v. 1.15.1)^65^, Biostrings (v. 2.42.1)^66^, data.table (v. 1.10.0)^67^, DESeq. 2 (v. 1.14.1)^68^, ggdendro (v. 0.1–20)^69^, and stringr (v. 1.1.0)^70^, and cowplot (v. 0.7.0). The samples were subsampled to an even depth of 10 000 reads per sample. Archaeal primers were not specific to the domain, so sequences not classified as Archaea were discarded and the count transformed to a fraction of the archaeal reads. Ratios were calculated between the average read abundance for a given OTU within the sample group (mesophilic digesters, thermophilic digesters, mesophilic digesters with thermal hydrolysis pre-treatment) and the average read abundance in the influent streams (primary and surplus sludge).

### Data availability

Amplicon sequencing data is available at the ENA with the project ID PRJEB15624. OTU tables and metadata files are available at figshare (DOI: 10.6084/m9.figshare.4308191). The RMarkdown files to generate the figures are available at github (github.com/Kirk3gaard/Publications/tree/master/Kirkegaard2017). The curated MiDAS taxonomy^32^ (v. 2.1) is available for download from the MiDAS website (midasfieldguide.org/en/download/).

## Electronic supplementary material

Supplementary info

Supplementary Table S1

Supplementary Table S2
