# Supplementary material for: The impact of immigration on microbial community composition in full-scale anaerobic digesters
Source: Sci Rep. 2017 Aug 24;7:9343. doi: 10.1038/s41598-017-09303-0 (PMC5571154; doi:10.1038/s41598-017-09303-0)
Supplement: Supplementary file 1 — Supplementary info [file 41598_2017_9303_MOESM1_ESM.pdf]

Supplementary:

The impact of immigration on microbial community composition in full-scale anaerobic digesters

Rasmus H. Kirkegaard, Simon J. McIlroy, Jannie M. Kristensen, Marta Nierychlo, Søren M. Karst, Morten S. Dueholm, Mads Albertsen and Per H. Nielsen.

Centre for Microbial Communities, Department of Chemistry and Bioscience, Aalborg University, Fredrik Bajers Vej 7H, DK-9220 Aalborg, Denmark.

*Correspondence to phn@bio.aau.dk

Figure S1

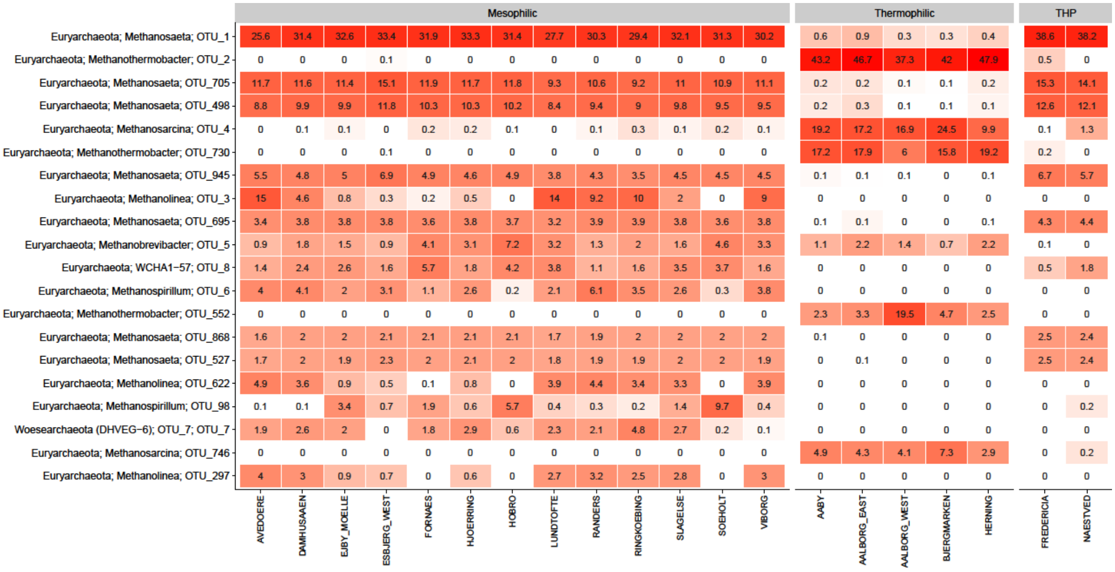

Figure S1| Heatmap of the 20 most abundant archaeal OTUs. Phylum and genus level classifications of the OTUs are shown, when no classification is available OTU number is given. The wastewater treatment plants had a total of 32 AD reactors (1-4 per plant) and they were analysed 2-23 times. The mean read abundance is shown for each plant. The taxa are sorted by mean read abundance across the plants.

Figure S2

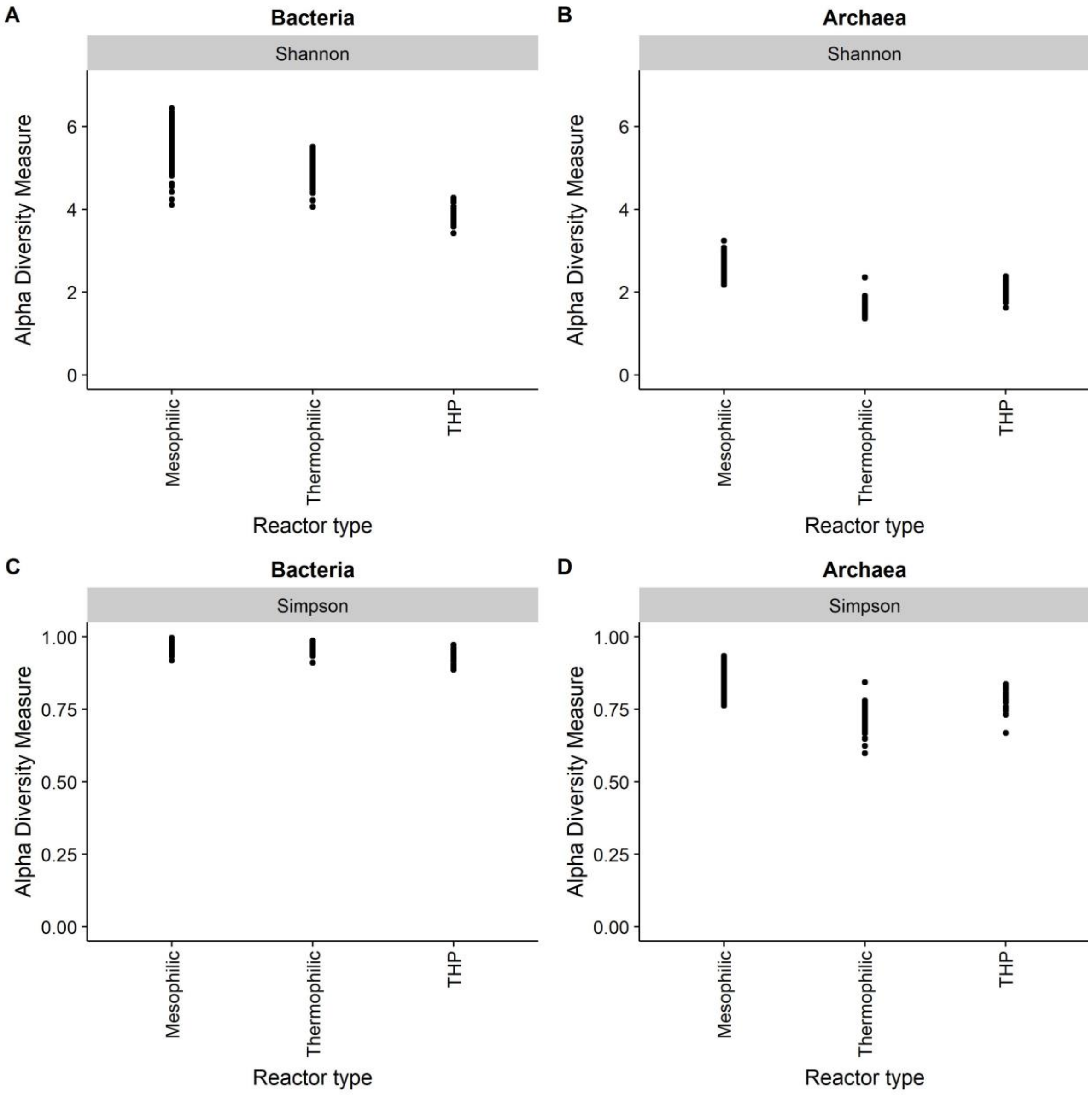

Figure S2| Alpha diversity indices. A) Shannon index for bacteria, B) Shannon index for archaea, C) Simpson index for bacteria, D) Simpson index for archaea.

Figure S3

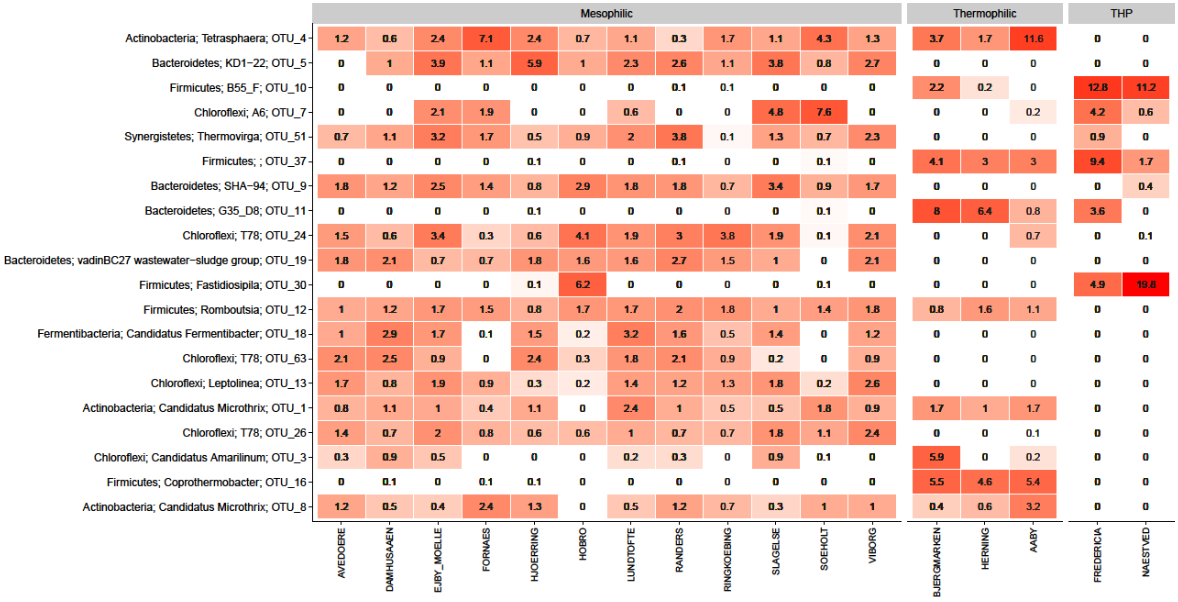

Figure S3| Heatmap of the 20 most abundant bacterial OTUs. Phylum and genus level classifications of the OTUs are shown, when no classification is available the field is empty. The wastewater treatment plants had a total of 32 AD reactors (1-4 per plant) and they were analysed 3-30 times. The mean read abundance is shown for each plant. The taxa are sorted by mean read abundance across the plants.

Figure S4

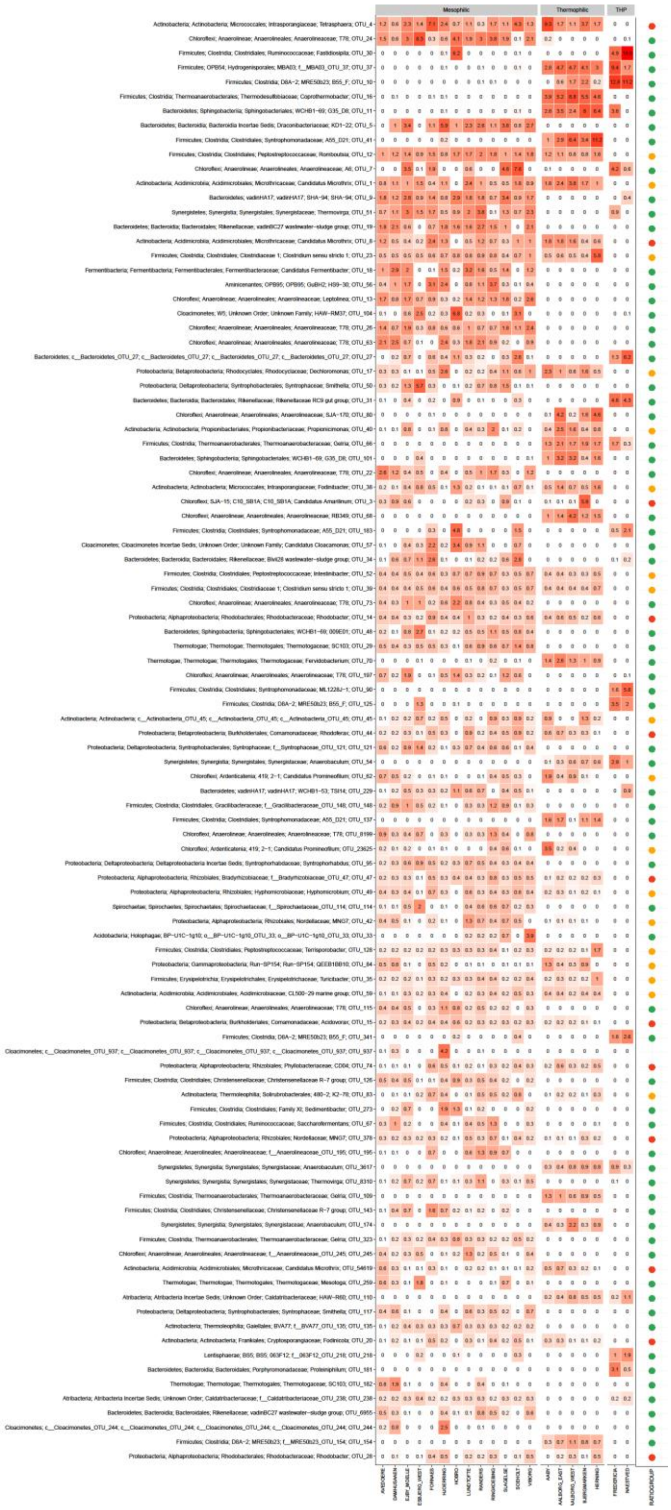

Figure S4| Heatmap of the 100 most abundant bacterial OTUs. The wastewater treatment plants had a total of 32 AD reactors (1-4 per plant) and they were analysed 3-30 times. The mean read abundance is shown for each plant. The taxa are sorted by mean read abundance across the plants. The right panel indicates whether the digester to influent read abundance ratio was above 10 (●) and likely actively growing, between 1 and 10 (●), or below 1 (●), indicating no growth.

Figure S5

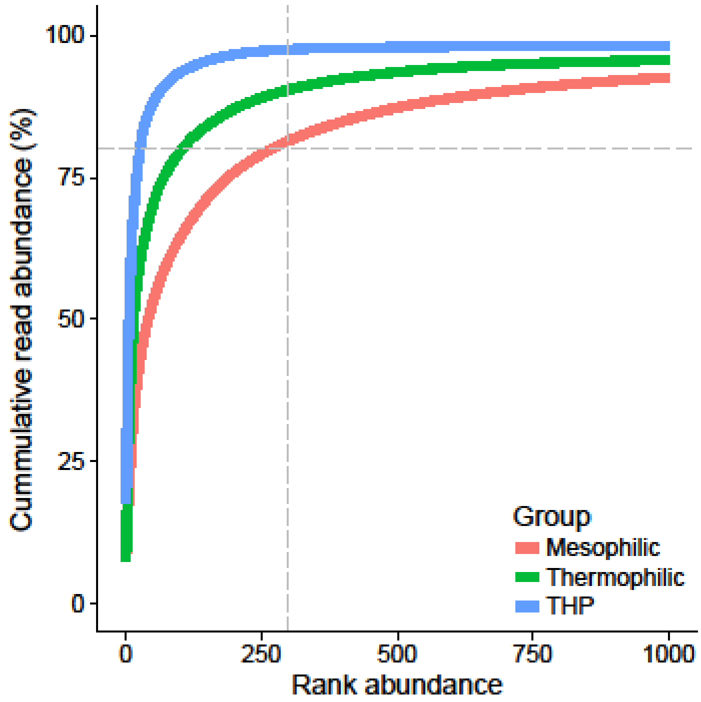

Figure S5| Rank abundance curve indicating the number of OTUs needed to account for a certain fraction of the cumulative reads for mesophilic (■), thermophilic (■) and mesophilic with THP (■) samples. Those constituting 80% are often defined as the abundant core community.

Figure S6

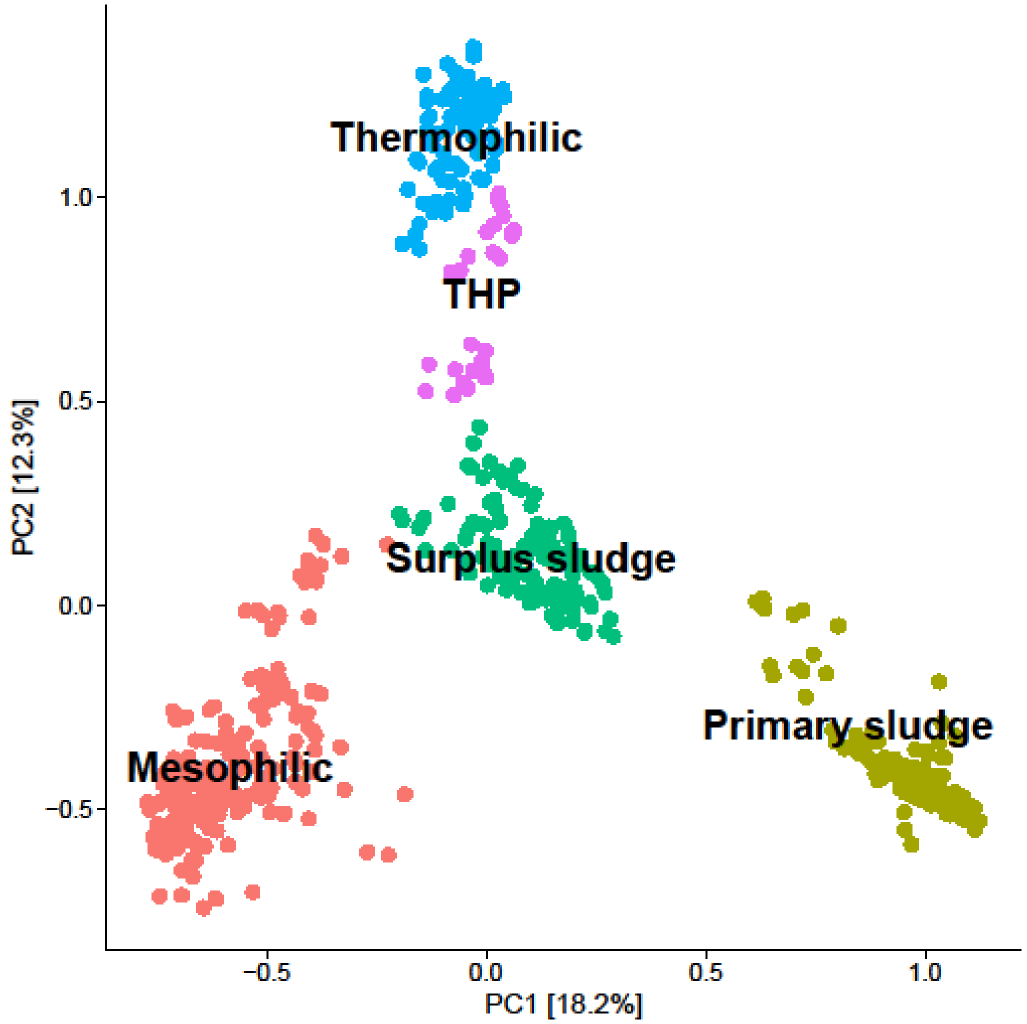

Figure S6| Principal component analysis of the bacterial communities analysed in this study highlighting samples by process type information. Mesophilic (●), thermophilic (●), and mesophilic with thermal hydrolysis pre-treatment (● THP), primary sludge (●) and surplus sludge (●).

Figure S7

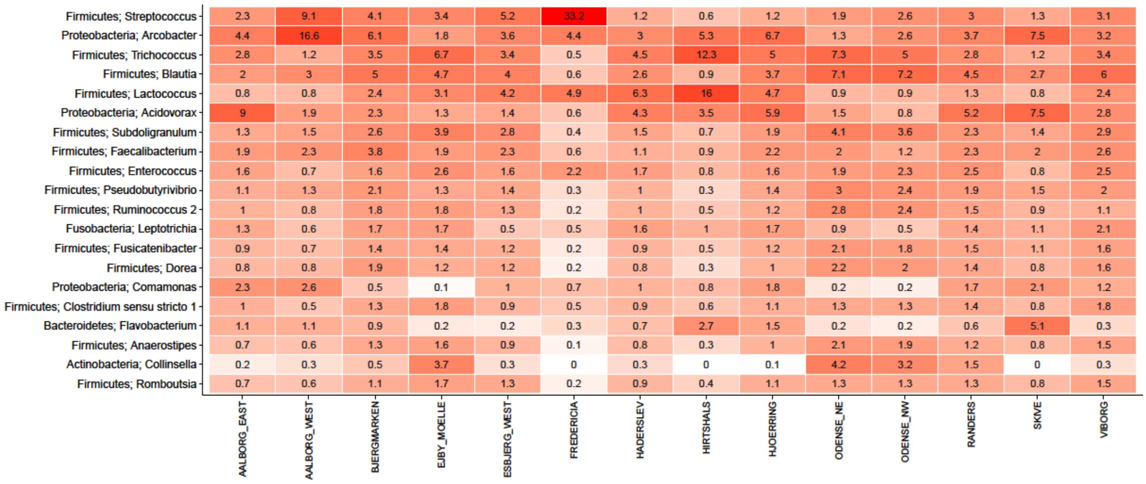

Figure S7| Heatmap of the 20 most abundant genera in the primary sludge. Phylum level classifications are shown. The mean read abundances are shown for each plant (3-23 samples per plant). The taxa are sorted by mean read abundance across the plants.

Figure S8

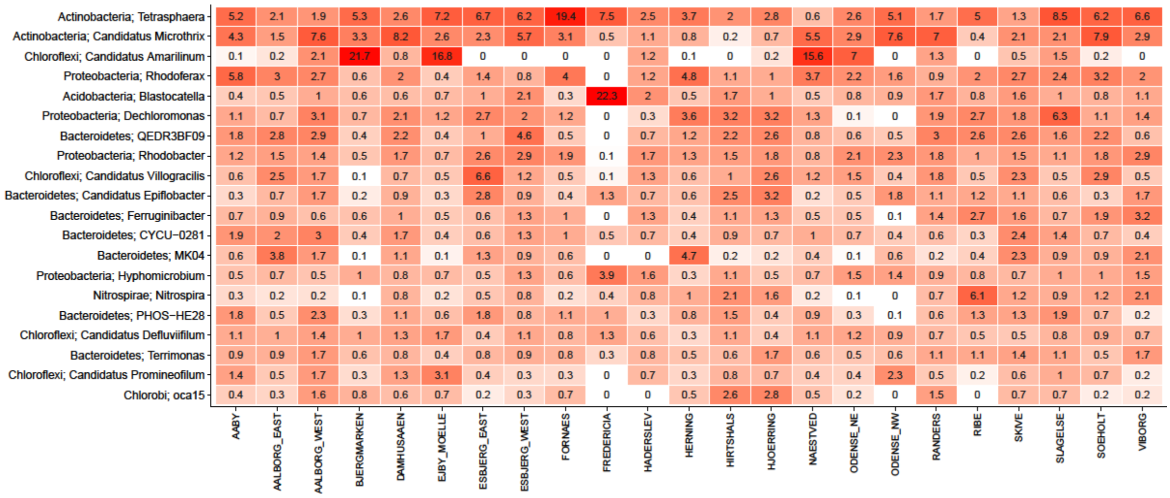

Figure S8| Heatmap of the 20 most abundant genera in the surplus sludge. Phylum level classifications are shown. The mean read abundance is shown for plants with more than 1 sample (1-24 samples per plant). The taxa are sorted by mean read abundance across the plants.

Figure S9

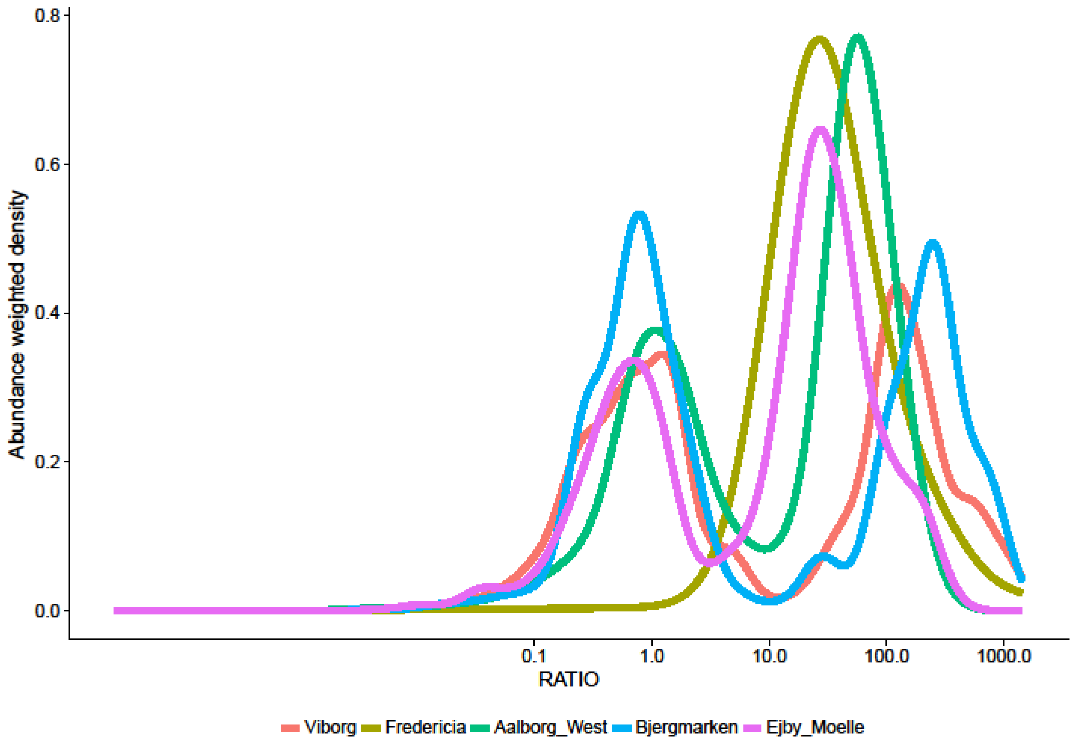

Figure S9| Density plot for ratios between OTU read abundance in the anaerobic digesters and the influent streams (primary and surplus sludge) weighted by the read abundance in the anaerobic digesters. Frederica has thermal hydrolysis pre-treatment.

Figure S10

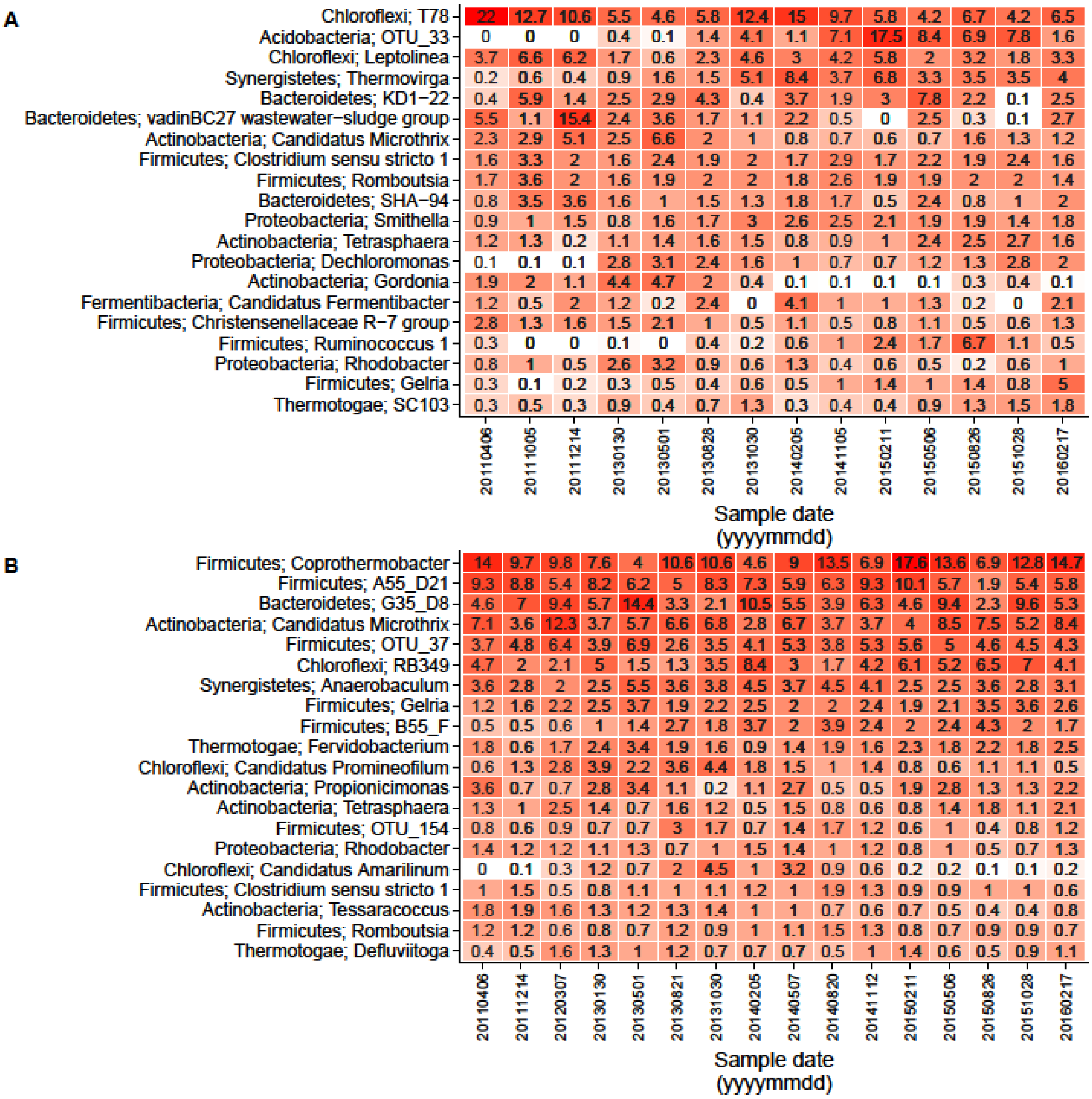

Figure S10| A) Heatmap of the 15 most abundant genera in the mesophilic reactor at Viborg during the survey period. B) Heatmap of the 15 most abundant genera in the thermophilic reactor at Aalborg West during the survey period.

Figure S11

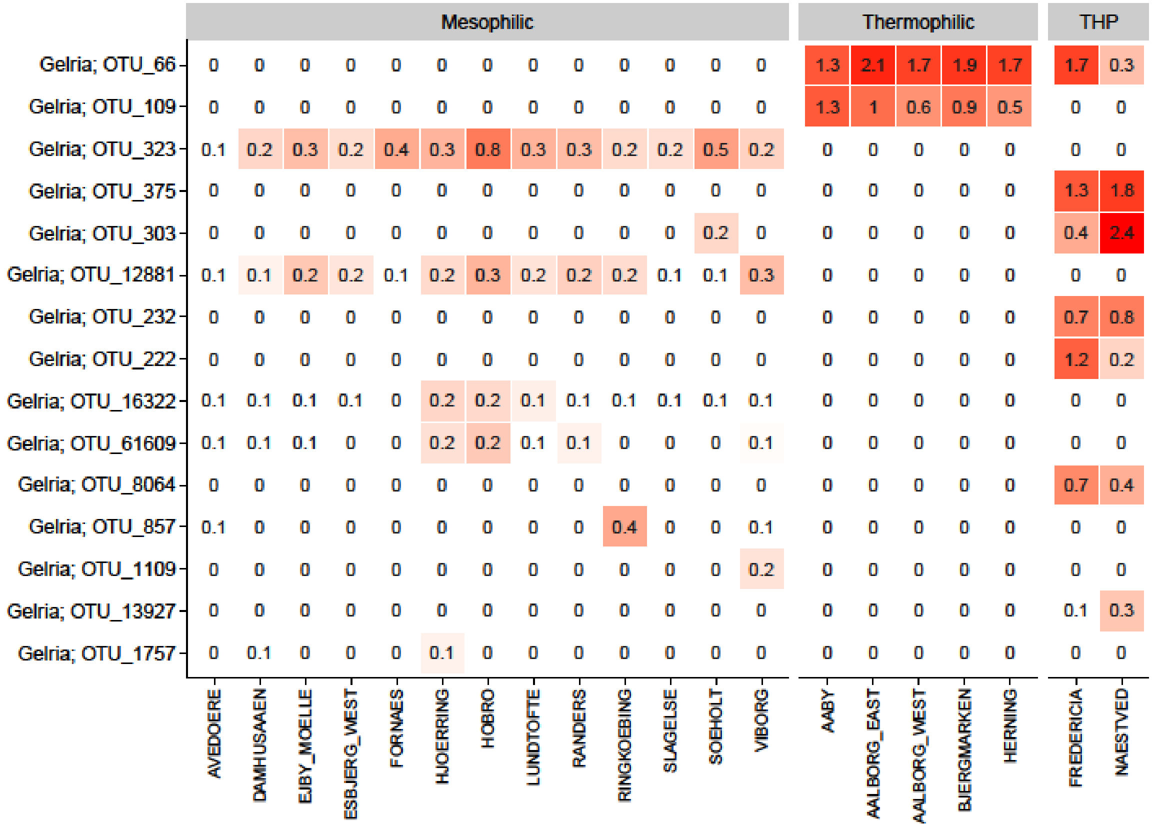

Figure S11| Heatmap of the 15 most abundant OTUs belonging to the genus *Gelria* in mesophilic and thermophilic systems. The wastewater treatment plants had a total of 32 AD reactors (1-4 per plant) and they were analysed 3-30 times. The mean read abundance is shown for each plant. The taxa are sorted by mean read abundance across the plants.

TableS1

Table S1 | Sample site information.

Uploaded as ".csv" file as it is not suitable for word

TableS2

Table S2 | Digester to influent ratios, and mean read abundance values at the OTU level for mesophilic, thermophilic and mesophilic with THP.

Uploaded as ".csv" file as it is not suitable for word
